# Linking hard and soft traits: Physiology, morphology and anatomy interact to determine habitat affinities to soil water availability in herbaceous dicots

**DOI:** 10.1371/journal.pone.0193130

**Published:** 2018-03-28

**Authors:** Michaël Belluau, Bill Shipley

**Affiliations:** Département de Biologie, Université de Sherbrooke, Sherbrooke (QC), Canada; Chinese Academy of Forestry, CHINA

## Abstract

**Background and aims:**

Species’ habitat affinities along environmental gradients should be determined by a combination of physiological (hard) and morpho-anatomical (soft) traits. Using a gradient of soil water availability, we address three questions: How well can we predict habitat affinities from hard traits, from soft traits, and from a combination of the two? How well can we predict species' physiological responses to drought (hard traits) from their soft traits? Can we model a causal sequence as soft traits → hard traits → species distributions?

**Methods:**

We chose 25 species of herbaceous dicots whose affinities for soil moisture have already been linked to 5 physiological traits (stomatal conductance and net photosynthesis measured at soil field capacity, water use efficiency, stomatal conductance and soil water potential measured when leaves begin to wilt). Under controlled conditions in soils at field capacity, we measured five soft traits (leaf dry matter content, specific leaf area, leaf nitrogen content, stomatal area, specific root length).

**Key results:**

Soft traits alone were poor predictors (R^2^ = 0.129) while hard traits explained 48% of species habitat affinities. Moreover, hard traits were significantly related to combinations of soft traits. From *a priori* biological knowledge and hypothesized ecological links we built a path model showing a sequential pattern soft traits → hard traits → species distributions and accounting for 59.6% (*p* = 0.782) of habitat wetness.

**Conclusions:**

Both direct and indirect causal relationships existed between soft traits, hard traits and species’ habitat preferences. The poor predictive abilities of soft traits alone were due to the existence of antagonistic and synergistic direct and indirect effects of soft traits on habitat preferences mediated by the hard traits. To obtain a more realistic model applicable to a population level, it has to be tested in an experiment including species competition for water supply.

## Introduction

Variation in soil water availability is one of the major environmental gradients along which plant species are differentially distributed [1] and is the environmental gradient of interest in this study. Presumably, the realized hydrological niches of such differentially distributed species are determined by both biotic interactions within the community and by trade-offs among functional traits involved in the water economy of a plant. It so then one should be able to predict both whole-plant performance and species relative abundances along environmental gradients given the relevant functional traits, but which traits should be used? Choosing the right traits is a key challenge in trait-based ecology and one that has not yet been completely met [2,3].

Functional traits can be grouped in ensembles possessing hierarchical relationships between them [4,5]. The group at the top of the hierarchy is formed by morpho-anatomical traits. These traits are less plastic and have slow response times to fluctuating environments compared to traits that are placed lower in the hierarchy, i.e., physiological traits. Physiological traits are constrained to a particular range of variation by the morphological and anatomical ones, but are more plastic and respond more quickly to changes in water status. Silvertown et al. (2015) [5] developed a similar idea with trade-offs in water economy at different organizational levels. At a morpho-anatomical level, the trade-off is related to the safety or efficiency of water transport. At a physiological level, the trade-off is related to leaf gas change. As we focus on traits related to environmental gradients, we are primarily interested in response traits. Those traits values change in response to environmental factors or variation applied to the community, by contrast with effects traits that act on the ecosystem processes. A given traits can be both a response and an effect trait [6].Another way to look at trait hierarchies is through the “hard” versus “soft” trait distinction [7–9]. “Hard” traits are those, usually physiological, which capture a precise function but are either difficult or expensive to measure. “Soft” traits, in contrast, are surrogates or proxies for such functions and are less difficult or expensive to obtain. “Soft” traits are usually morphological or anatomical. Given the previously described hierarchy one would expect that the causal relationships should follow the order: soft→hard→performance. Since environmentally-dependent plant performance (growth, survival, reproduction) is a key component determining habitat affinities and population distributions, the predictions of habitat affinities should be more robust when using the indirect path (soft→hard→habitat) than when using the direct path (soft→habitat).

**Fig 1:**
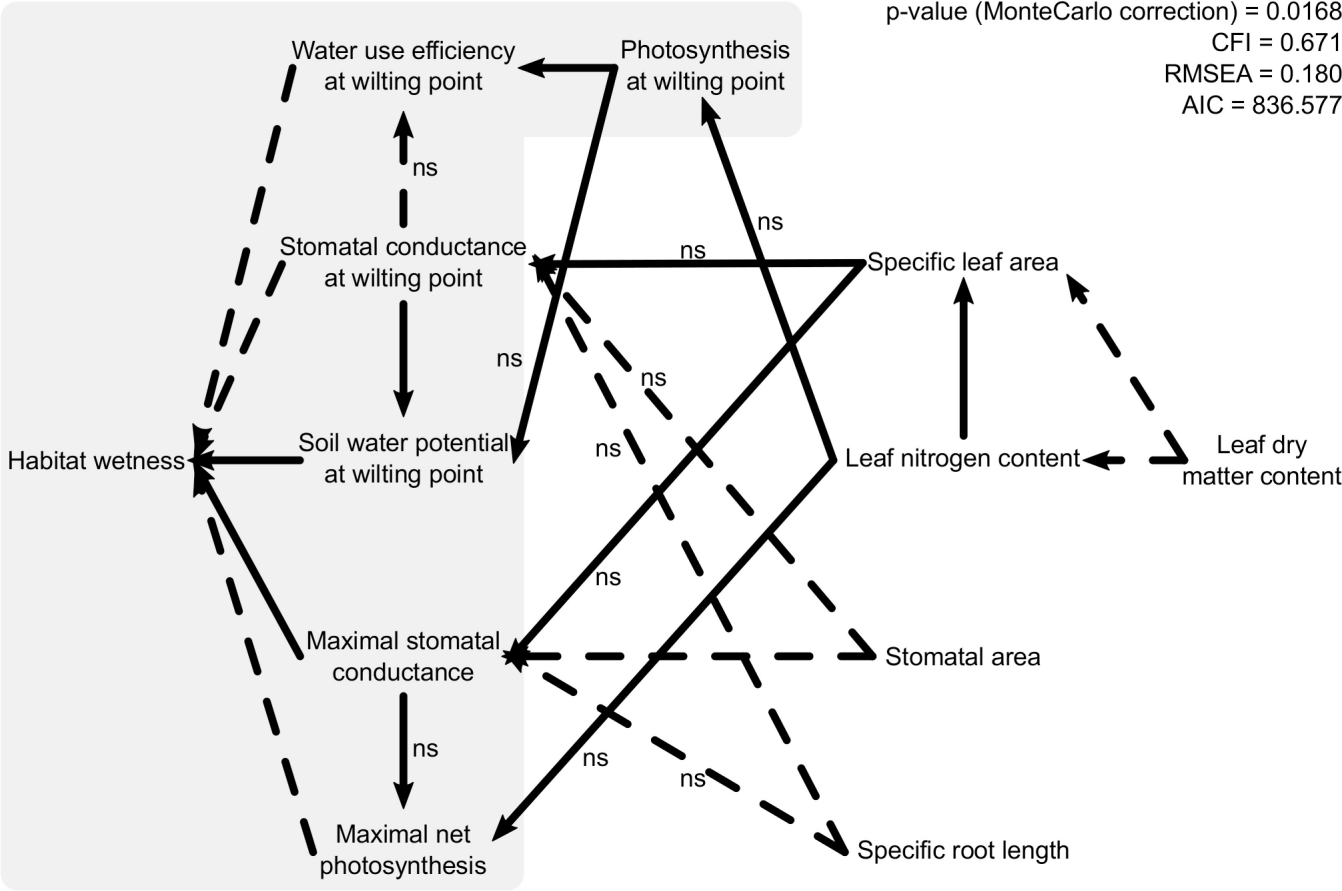
Initial (hypothetical) path model of the physiological and morphological traits. The grey part is the path model from Belluau and Shipley (2017) [10]. Solid lines are positive causal relationships while dashed lines are negative. The path coefficients are not reported here except for non-significant links (ns). This initial (hypothetical) path model was statistically rejected (Satorra-Bentler robust Chi-square = 85.051; 47 df; *p* = 0.0168; Comparative Fit Index (CFI) = 0.671; Root Mean Square Error of Approximation (RMSEA) = 0.180).

We tested this prediction by building on the study of Belluau and Shipley (2017) [10]. They used 25 herbaceous species differing in their field distribution along a gradient of soil water availability and took a series of physiological traits related to leaf gas exchange and leaf wilting during an experimental period of drought. They identified five physiological “hard” traits that collectively predicted the known soil moisture affinities of each species: (i) water use efficiency at the first clear wilting stage (WUE_wilt_); (ii) maximal stomatal conductance at soil field capacity (g_s_^max^); (iii) soil water potential at the first clear wilting stage (Ψsoil_wilt_); (iv) maximum net photosynthesis (A_max_, i.e., at soil field capacity); and (v) stomatal conductance at the first clear wilting stage (g_s_^wilt^). These traits are time-consuming to measure and require experimental manipulations of soil water availability. Normally, one would want to predict ecological responses using easily measurable traits in the field and under normal (i.e. non-drought) conditions. In this study we introduce a series of static morpho-anatomical (“soft”) traits measured on plants of the same species but growing under non-drought conditions. Using this series of soft and hard traits, we asked three questions:

1. How well can we predict habitat affinities only from hard traits, only from soft traits, or from a combination of the two? Do we lose predictive ability of habitat affinities (a demographic consequence) by going directly from soft (morphological) traits to species distributions?
2. How well can we predict species' physiological responses to drought (hard traits) from their morphological traits (soft traits)?
3. Can we model the causal sequence as soft traits → hard traits → species distributions as shown in the multivariate hypothesis summarized in Fig 1?

## Material and methods

### Experimental design

#### Study species and habitat affinities

We used 25 species of dicots from 18 different families (Table 1). All species were herbs, except for *Cistus salvifolius*, a small shrub which does not produce lignified tissues during its first year and so is a functional herb during this period. These species were selected (i) to represent all three hydrological stages along a gradient of affinities for water from dry to wet soils while excluding the extremes (deserts and aquatic habitats) of the hydrological gradient, (ii) to represent every phenological strategy of herbs (annual, biannual and perennial) and (iii) to cover the range of functional traits and adaptive strategies that are described and quantified by Grime et al. (2007) [11]. We selected species based on an ordinal ranking of habitat affinity giving 11 species typical of dry soils (hereafter "dry"), 9 species typical of intermediate soils (hereafter "intermediate") and 5 species typical of wet soils (hereafter "wet"); see Belluau and Shipley (2017) [10] for details on how habitat affinities were assigned. To assign a species to a given ordinal rank, we combined several trusted sources, mainly floras and USDA databases (details about the sources are reported in Belluau and Shipley (2017) ([10], Supplementary Information). An ordinal variable is a categorical variable for which the possible values are ordered (here, the level of soil wetness) but are unitless and not quantitative. This type of data requires a special set of statistical analysis, the cumulative link model described in Belluau and Shipley (2017) [9] and applied in Shipley et al. (2017) [12].

**Table 1:** Species list containing soil hydrological classification, life cycle, orders and families.

Species name	Soil hydrology	Life cycle	Order	Family
*Achillea millefolium*	Dry	Perennial	Asterales	Asteraceae
*Asclepias syriaca*	Dry	Perennial	Gentianales	Apocynaceae
*Campanula americana*	Intermediate	Annual / Biannual	Asterales	Campanulaceae
*Centaurea cyanus*	Intermediate	Annual / Biannual	Asterales	Asteraceae
*Cerastium tomentosum*	Dry	Perennial	Asterales	Caryophyllaceae
*Chenopodium polyspermum*	Wet	Annual	Caryophyllales	Amaranthaceae
*Cistus salviifolius*	Dry	Perennial	Violales	Cistaceae
*Daucus carota*	Intermediate	Biannual	Apiales	Apiaceae
*Epilobium ciliatum* subsp. *Glandulosum*	Wet	Perennial	Myrtales	Onagraceae
*Erigeron canadensis*	Dry	Annual	Asterales	Asteraceae
*Eupatorium perfoliatum*	Wet	Perennial	Asterales	Asteraceae
*Hypericum perforatum*	Wet	Perennial	Malpighiales	Hypericaceae
*Jacquemontia gracillima*	Intermediate	Perennial	Solanales	Convolvulaceae
*Lythrum salicaria*	Wet	Perennial	Myrtales	Lythraceae
*Medicago lupulina*	Dry	Annual / Biannual	Fabales	Fabaceae
*Medicago sativa*	Intermediate	Perennial	Fabales	Fabaceae
*Melilotus albus*	Dry	Biannual	Fabales	Fabaceae
*Myagrum perfoliatum*	Intermediate	Annual	Brassicales	Brassicaceae
*Nigella damascena*	Intermediate	Annual	Ranunculales	Ranunculaceae
*Plantago lanceolata*	Dry	Perennial	Lamiales	Plantaginaceae
*Rumex acetosella*	Dry	Perennial	Caryophyllales	Polygonaceae
*Scorzoneroides autumnalis*	Dry	Perennial	Asterales	Asteraceae
*Silene vulgaris*	Dry	Perennial	Asterales	Caryophyllaceae
*Stellaria media*	Intermediate	Annual	Asterales	Caryophyllaceae
*Trifolium pratense*	Intermediate	Perennial	Fabales	Fabaceae

Taxonomic nomenclature follows The International Plant Names Index (2016) http://www.ipni.org [accessed 14/11/2016]. The soil hydrology classification of the species is based on several sources reported in Belluau and Shipley (2017, [Supporting information]) [10] along with taxonomic authorities.

#### Experimental design

Plants were grown under controlled conditions. Photosynthetically active photon flux was 160 μmol m^-2^ s^-1^ ± 20 μmol m^-2^ s^-1^ PAR using a 1000W high-pressure sodium and a 1000W metal-halide bulb for 12 hours per day, giving a Daily Quantum Yield of 6.912 mol m^-2^ day^-1^. These values are lower than typically encountered in the field but are standard in experiments conducted in growth chambers and are sufficient to insure normal growth rates [13]. Daytime temperature was 24°C (± 2°C), and was decreased to 20°C (± 2°C) at night. Relative humidity (RH) of the air was 45% (± 5%). The planting substrate was 90% sand and 10% peat loam, with an additional 6 g of NPK fertilizer (7-12-12) per kg of soil. These growing conditions are the same as Belluau and Shipley (2017) [10]. Each species was represented by 5 individual plants, planted in separate pots (10x10x22deep cm^3^, approximatively 2L per pot). Seeds were germinated directly in the pots. Individuals per species were selected to be most similar in terms of age, height and number of leaves at the beginning of the measurements. The position of each pot was fully randomized. All plants were grown in soil that was constantly maintained at field water capacity during the experiment by adding sufficient water every two days. Volumetric water content was estimated from soil dielectric permittivity measurements (HydroSense II probe, Campbell Scientific, Edmonton, AB, Canada). The probe was placed in the centre of the pot to avoid edge effects. Measurements begin when the plants reached a size, between two and four fully grown leaves) and an age of approximatively one month, size and age comparable with the plants used in the previous experiment reported in Belluau and Shipley (2017) [10].

### Measurements

#### Morphological and anatomical traits

We measured traits on leaves and roots of each species. All traits in this study were measured using the protocols found in Cornelissen et al. (2003), Pérez-Harguindeguy et al. (2013) [2,14] and PrometheusWiki web site ([15]; http://prometheuswiki.publish.csiro.au/tiki-custom_home.php) and are reported in Table 2.

**Table 2:** List and description of the soft and hard traits used in the analysis.

Traits	Symbol	Unit	Description
*(A) Soft traits used in the present path analysis*			
*Leaf dry matter content*	LDMC	g g^-1^	Leaf dry matter content at field capacity, calculated from all mature leaves fresh weight divided by all mature leaves dry weight.
*Specific leaf area*	SLA	m^2^ kg^-1^	Specific leaf area at field capacity, calculated as the ratio between the leaf area of all mature leaves divided by the dry mass of all matures leaves.
*Leaf nitrogen content*	LNC	mg g^-1^	Leaf nitrogen content is calculated as the dry mass of nitrogen on the dry mass of the entire sample.
*Stomatal area*	Stom_*area*_	μm^2^	Average stomatal area, calculated from the average length and width of guard cells.
*Specific root length*	SRL	m g^-1^	Specific root length at field capacity, calculated as the ratio between the root length of half of the root system to the dry mass of the same half of the root system.
*(B) Hard traits from Belluau & Shipley (2017)* [10]			
*Maximum stomatal conductance*	g_*s*_^*max*^	mmol_H2O_ m^-2^ s^-1^	Stomatal conductance at field capacity, calculated from the piecewise regression.
*Stomatal conductance at stage 2 wilting point*	g_*s*_^*wilt*^	mmol_H2O_ m^-2^ s^-1^	Stomatal conductance at the stage 2 wilting point, based on the average volumetric water content at stage 2 wilting point (water volume/soil volume, 100 x m^3^.m^-3^), calculated from the piecewise regression.
*Soil water potential at stage 2 wilting point*	Ψ_*wilt*_	MPA	Soil water potential at stage 2 of visual wilting point, calculated from the equation relating volumetric water content and water potential.
*Maximum net photosynthesis*	A_*max*_	μmol_co2_.m^-2^.s^-1^	Net photosynthesis at field capacity, the first day of experiment.
*Net photosynthesis at stage 2 wilting point*	A_*wilt*_	μmol_co2_.m^-2^.s^-1^	Net photosynthesis measured when the individual reach the stage 2 wilting point.
*Water use efficiency at stage 2 wilting point*	WUE_wilt_	μmol_CO2_.m^-2^.s^-1^ / mmol_H2O_.m^-2^.s^-1^	Water use efficiency calculated when the individual reach the stage 2 wilting point, based on Net photosynthesis at stage 2 wilting point.

(A) The five soft traits are the morphological traits predicting hard traits, identified in the path analysis (Fig 2). (B) The six hard traits are the physiological traits identified as predictors of the habitat affinity to soil wetness, identified in Belluau & Shipley (2017) [10]. Mean traits values for all 25 species are reported in S1 Table.

Leaf dry matter content (LDMC, g.g^-1^) is measured as the ratio between the dry mass on the fresh mass of all mature leaves of the entire individual. Specific leaf area (SLA, m^2^ kg^-1^) is measured as the ratio between the leaf area on the dry mass of all mature leaves of the entire individual. Leaf area was measured using a scanner. Leaf nitrogen content (LNC, mg g^-1^) is measured as the ratio between the dry mass of leaf nitrogen to the dry mass of the leaf. Leaf nitrogen mass was analysed by dry combustion (Vario Macro, Elementar analysensystem GmbH, Haan, Germany). Stomatal area (Stom_area_, μm^2^) was calculated by approximating the stomate as an ellipse: area = (π/2) (stomatal length) (stomatal width). Leaf imprints were obtained by placing clear nail polish on the underside of a leaf and then taking digital images from a microscope using an objective lens at 20X and 40X, representing a field of vision of approximately 0.307 and 0.080 cm^2^ respectively (Microscope: Axio Observer Z1; Lens: Zeiss 20x/0.8 Plan-Apochromat, Zeiss 40x/0.95 Plan-Apochromat: Camera: Zeiss Axiocam 506 mono; Carl Zeiss AG, Oberkochen, Germany). Stomatal length and width (μm) were measured using the imaging software Fiji [16]. The length and width measured are the guard cells maximum length and width. Measures were taken on five stomates per image, ten images per leaf and one leaf from each two individuals per species for a total of 100 stomates per species. The pictures are taken along both sides of the midrib in the middle of the leaf. We used the average value of all measurements per individual per species. Specific root length (SRL, m.g-1) was measured as the ratio between the root length of half of the root system to the dry mass of the same half of the root system. After cleaning of the root system, the root length was measured using a scanner and the WinRhyzo software (Régent Instruments Inc., Québec, Canada).

### Data analysis

#### Predictive ability using a cumulative link model analysis

Since the dependent variable (i.e. a species habitat affinity along the gradient of soil moisture) was in the form of an ordered category with three states (dry, intermediate or wet), while the independent variables were the trait values of each species, the proper statistical model is a cumulative link model (clm function in the *ordinal* package of R, [17]), which is an extension of logistic regression but made to analyse ordinal dependent observations having more than two states [18]. We use McFadden’s R^2^ [19], based on the ratio of the log-likelihood of the model to the log-likelihood of a model without the traits (i.e. an intercept-only model) to assess the predictive ability of the resulting model. This R^2^ is interpreted in the same way as a classic model R^2^ and reduces to the classic R^2^ (i.e. proportion of the variance explained by the model) in the case of a linear model with a normally distributed dependent variable.

#### Stepwise backward model selection by AIC

In order to explore the relationships between hard and soft traits, we conducted a stepwise backward model selection by the Akaike information criterion using the stepAIC function in the R package *MASS* [20]. This analysis was performed on each of the hard traits regressed on the five soft traits in a stepwise backward fashion (hard traits ~ five soft traits). We explore the hypothesis of a hierarchical relationship between physiological and morpho-anatomical traits [5]. This hypothesis state that the range of variation of the physiological traits is constrained by the morpho-anatomical ones, and not the reverse. We then constructed our model in the direction of soft traits predicting hard traits predicting habitat. Models were selected based the lowest value of AIC.

#### Path analysis

We used path analysis [21] to develop and test hypotheses concerning the causal relationships between the morphological traits, the physiological traits and the field distributions. Our initial hypothesized causal structure (Fig 1) reflects a combination of *a priori* definitional and ecological links and was subsequently modified to obtain a final model that successfully accounted for the patterns of conditional dependencies in the data. The modifications were made to improve the fit between the data and the model, conditional on such modifications not contradicting known biology. We used the *sem* function in the *lavaan* package of R [22]. We present the path coefficients based on standardized values with units of standard deviations of the mean in order to compare the relative importance of each variable. We used the maximum likelihood estimator (“MLM”) with standard errors and a means-adjusted (or “Satorra-Bentler”) Chi-square test statistic [23], both of which are robust to non-normality. We corrected the asymptotic null probabilities for our sample size using Monte Carlo methods ([21], p. 178). We conducted all statistical analyses within the R statistical environment ([24]; Version 3.1.2, R Core Development Team, 2015-12-04).

## Results

### Predictive ability of hydrological groups using soft versus hard traits

When considering each of the five soft traits separately, very few significant differences were detected between the mean morphological traits of the three groups of species based on their habitat preferences. Fig 2 presents boxplots of the five soft traits measured at field capacity according to groups of habitat wetness. Non-parametric (Kruskal-Wallis) 1-way ANOVAs did not detect any significant differences between the three species groups for any of the five traits. All traits values for all 25 species are reported in S1 Table.

**Fig 2:**
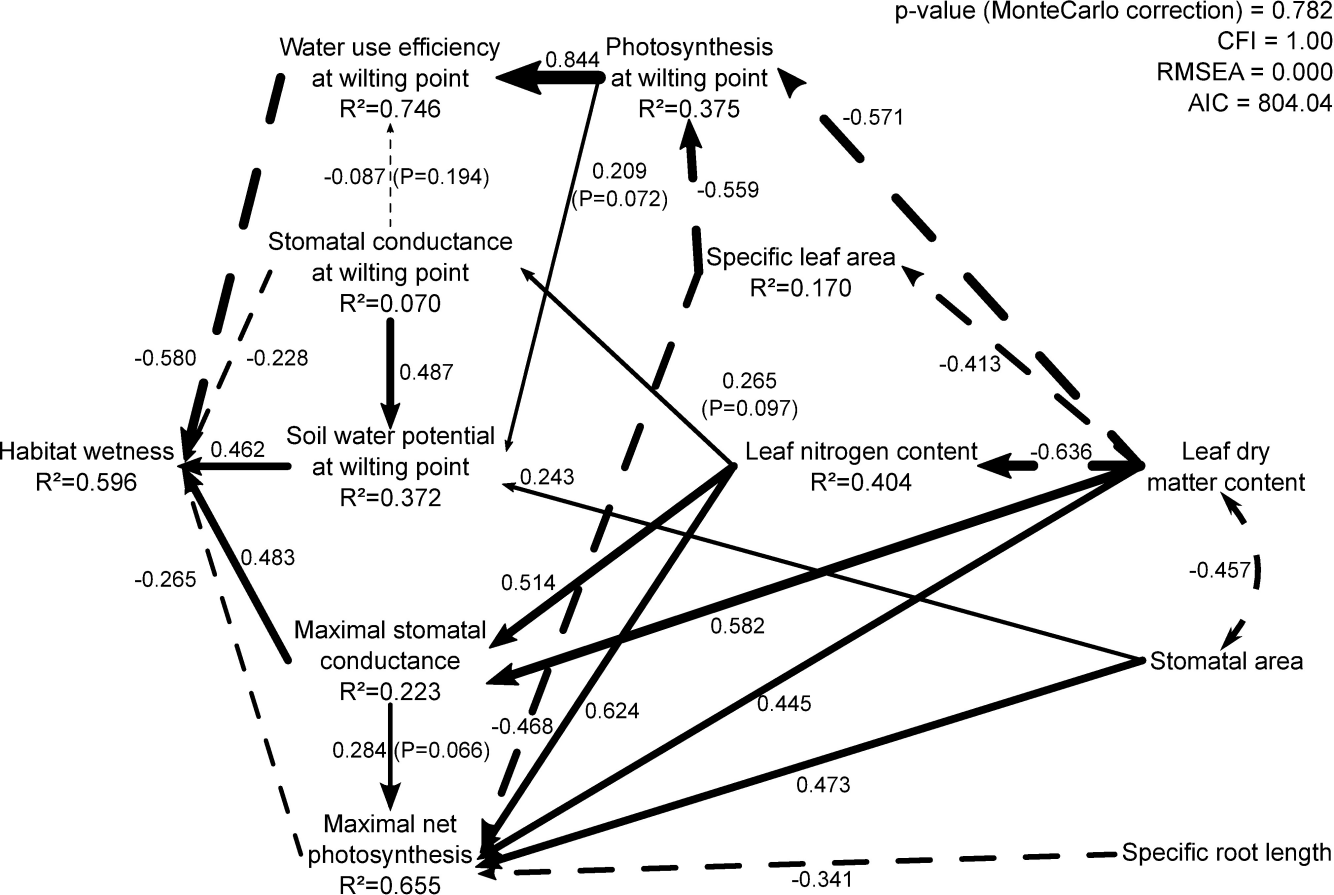
Path analysis of the physiological and morphological traits. There is no significant misfit between the empirical data and the causal structure specified by the model (Satorra-Bentler robust Chi-square = 40.795; 42 df; *p* = 0.782; Comparative Fit Index (CFI) = 1.00; Root Mean Square Error of Approximation (RMSEA) = 0.00), Akaike information criterion (AIC) = 804.037. All path coefficients are significantly different from zero, otherwise the paths’ significativity are specified in the diagram. The R^2^ are the percentage of variance explained by the causal variables. Values on the lines are the path coefficients between the causal variable and the caused variable. Solid lines are positive causal relationships and dashed lines are the negative ones. Thickness of the lines is proportional to the strength of path coefficients. For all traits, we used the average value of five individuals per species. For all leaf traits, we excluded the petioles because petioles don’t belong to the same function as the leaf blade (i.e. support for petioles and acquisition for leaf blade). Leaves were chosen to be representative of the average mature leaf into one individual and we avoided newly formed leaves or ones showing any sign of senescence.

Even in a multivariate context, soft traits alone were poor predictors of species habitat affinities. When regressing the five soft (i.e. morphological) traits together on the habitat affinities using the CLM analysis, only one trait (SLA) was significant (p = 0.031) (Table 3A) and these five soft traits together accounted for only 12.9% of the deviance in the habitat affinities of the species. This contrasts with the predictive ability of the hard traits (WUE_wilt_, g_s_^max^, g_s_^wilt^, A_max_ and Ψ_wilt_). When regressing these five physiological traits on the habitat affinities using a CLM analysis they were all statistically significant and together accounted for 48% of the deviance in the habitat affinities of the species (Table 3B).

**Table 3:** Cumulative link model analysis of the hard and soft traits as predictors of the habitat wetness preferences.

	Estimate	Std. Error	z value	Pr(>|z|)	
*(A) CLM with five soft traits used in the analysis*					
Specific leaf area	0.0814	0.038	2.157	0.031	*
Stomatal Area	-0.0033	0.002	-1.423	0.155	
Leaf nitrogen content	-0.0559	0.057	-0.983	0.325	
Leaf dry matter content	-2.5420	13.430	-0.189	0.850	
Specific root length	-0.0001	0.007	-0.012	0.990	
Threshold dry|medium	0.013	4.232	0.003		
Threshold medium|wet	2.018	4.247	0.482		
R^2^ (McFadden’s R^2^)	0.129				
*(B) CLM with five hard traits from Belluau and Shipley (20170)* [10]					
Water use efficiency at wilting point	-155.366	55.110	-2.819	0.005	**
Maximum conductance	0.023	0.009	2.696	0.007	**
Soil water potential at wilting point	0.506	0.220	2.304	0.021	*
Conductance at wilting point	-0.019	0.009	-2.039	0.041	*
Maximum Net Photosynthesis	-0.450	0.242	-1.862	0.063	.
Threshold dry|medium	-5.248	3.087	-1.700		
Threshold medium|wet	-1.603	2.707	-0.592		
R^2^ (McFadden’s R^2^)	0.481				

(A) With the five soft traits identified in the present path analysis explaining 12.9% of total deviance. (B) With the five hard traits from Belluau and Shipley (2017) [10] explaining 48.1% of total deviance.

### Predictive ability of hard traits using soft traits

The soft traits had variable success in predicting the hard traits using backwards stepwise regression (Table 4). Net photosynthetic rates and stomatal conductance at the start of the drought period and net photosynthetic rates at leaf wilting were significantly related to combinations of the soft traits while the other hard traits were not.

**Table 4:** Stepwise backward linear regression, based on AIC values, of each of five hard traits (A_wilt_, gswilt, Ψ_wilt_, gsmax, A_max_) on a linear combination of five soft traits (leaf dry matter content (LDMC, g g^-1^), specific leaf area (SLA, m^2^ kg^-1^), leaf nitrogen content (LNC, mg g^-1^), stomatal area (stom_area_, (μm) and specific root length (SRL, m g^-1^)).

Hard traits	Selected model	R_adj_^2^ (selected model)
A_wilt_	A_wilt_ ~ LDMC + SLA	0.3177
g_s_^wilt^	g_s_^wilt^ ~ none	0.0299
Ψ_*wilt*_	Ψ_wilt_ ~ none	0.1179
g_s_^max^	g_s_^max^ ~ LDMC + LNC	0.1521
A_max_	A_max_ ~ LDMC + SLA + LNC + stom_area_ + SRL	0.4210

Shown are those soft variables that are significant predictors (p<0.05) and the selected model adjusted R^2^.

### Testing hypothesized causal relationships between hard traits, soft traits and habitat affinities

Our initial causal hypothesis linking soft traits, hard traits and habitat affinities (Fig 1) was rejected (Satorra-Bentler robust Chi-square = 85.051: 47 df: *p* = 0.0168). We therefore modified this initial model to produce a second model (Fig 2) that shows no significant misfit between the empirical data and the causal structure (Satorra-Bentler robust Chi-square = 40.795; 42 df: *p* = 0.782). These modifications were guided by three principles: First, path coefficients in the initial model (Fig 1) that were not statistically different from zero were set to zero (i.e. removed) unless they had clear biological justifications. We maintained four such marginally non-significant path coefficients (shown in Fig 2). Second, we added new paths to our initial model if these were both biologically justifiable and were statistically significant (based the stepwise backward model selection above, Table 4). These added paths were (i) from LDMC to A_wilt_, A_max_ and g_s_^max^, (ii) from SLA to A_wilt_ and A_max_, (iii) from LNC to g_s_^wilt^ and g_s_^max^, (iv) from stomatal area to Ψ_wilt_, and A_max_ and (v) from SRL to A_max_. Finally, since the modified model required a link between stomatal area and LDMC although there was no clear biological justification for a causal link between them, we allowed these two variables to freely covary. The resulting path model accounted for 59.6% of the variation in the habitat wetness variable.

## Discussion

From Belluau and Shipley (2017) [10] we already know that the five physiological (hard) traits can predict the habitat affinities of our species along a gradient of soil water availability and that a plausible multivariate causal hypothesis linking them is consistent with both the observed data a pre-existing knowledge. However, these traits are difficult and time-consuming to measure and some of them require that the plant already be wilting before measurements are taken. This limits their usefulness in large-scale trait-based ecology. We expected, given the hierarchy of traits described by Chapin III, Autumn and Pugnaire (1993) and Silvertown et al. (2015) [4,5] that (i) soft (morpho-anatomical) traits would be more loosely linked to habitat affinities than the hard traits; that (ii) the soft traits would, none the less, link to the hard traits; and that (iii) we should be able to express the links as soft→hard→habitat.

### Soft traits alone don’t predict habitat preferences

**Fig 3:**
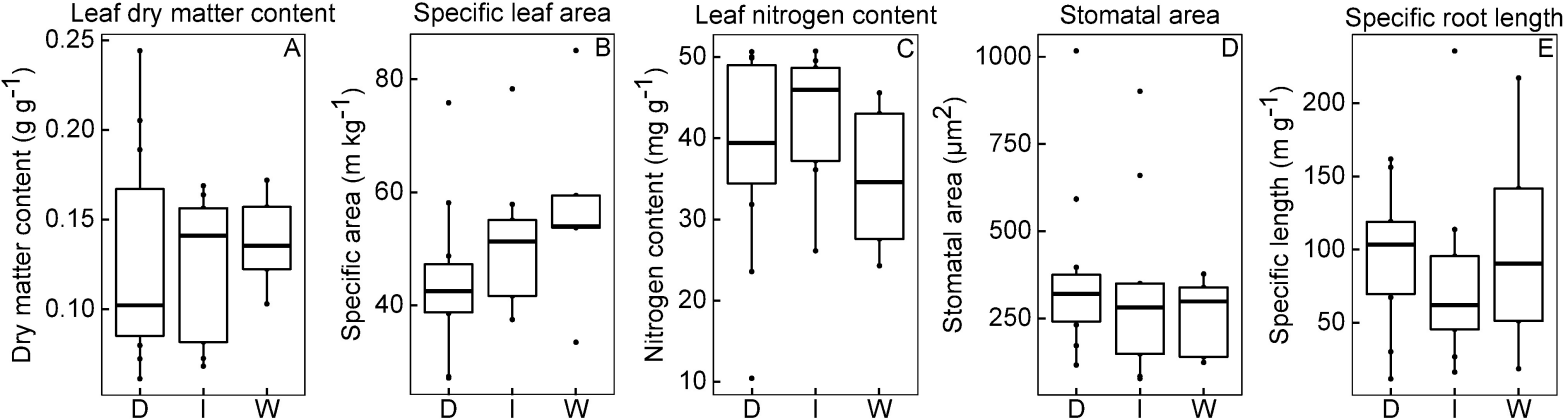
Box plots showing the differences between species means of the soft trait values. Traits are measured at field capacity and grouped by species according to affinity for habitat wetness. "D" (species typical of "dry" soils), "I" (species typical of "intermediate" soils), "W" (species typical of "wet" soils). Non-parametric (Kruskal-Wallis) 1-way ANOVAs did not detect any significant differences between the three species groups for any of these five traits.

Our first expectation was confirmed. The soft traits were more loosely linked to habitat affinities that the hard traits. First, the trait values of the “dry”, “intermediate” and “wet” species were largely overlapping (Fig 3) and no significant differences between these three groups were detected for any single soft trait. In fact, only SLA was significantly related to the different habitat affinities and explained only 13% of the variance explained (Table 3A). This contrasts with the CLM analysis done on hard traits Belluau and Shipley (2017) [10] which explained 48.1% of the variance in the habitat affinities (Table 3B). Despite the weak correlation between the soft traits and the habitat affinities, this does not necessarily mean that no link exists. Those links could be indirect or very loose.

### Can species' hard traits be predicted from their soft traits?

Our second expectation, that the physiological traits could be linked to the morpho-anatomical traits measured in the absence of stress, was only partly confirmed when looking only at the regression results. Some hard traits have relationships with soft traits while other don’t. However, the final path model (Fig 2, discussed further below) provided an explanation for this.

Regarding net photosynthetic rates and stomatal conductance at the start of the drought period and net photosynthetic rates at leaf wilting, the literature largely confirms the relationships identified in the stepwise analysis (Table 4 and S2 Table). Since soft traits are by definition surrogates or proxies of functions in the plant, it is not surprising to find relationships between stomatal conductance and photosynthesis at field capacity, and common leaf traits. Those relationships are foundation stones of the functional ecology approach [9]. In the stepwise analysis, maximum stomatal conductance (g_s_^max^) is related to LNC and LDMC [25–28]. In the same fashion, maximum net photosynthesis (A_max_) is well related to all soft traits [29–33]. Considering A_wilt_, we could expect that the relationships existing a field capacity are conserved in drought conditions [29,30]. This expectation is confirmed by the stepwise analysis and by the literature [34]. However, stomatal conductance at wilting (g_s_^wilt^) and soil water potential at wilting (Ψ_wilt_) were not related to any soft traits even if we could have expected correlations between g_s_^wilt^ and LNC and between Ψ_wilt_, and stom_area_ considering the literature [25,27,35,36].

The path analysis allows us to explore the relationships between hard and soft traits detected by the stepwise analysis as well as the relationship between soft traits. Some non-significant relationships in the stepwise analysis can be biologically and statistically relevant in a path analysis.

### Direct and indirect relationships between soft traits, hard traits and habitat preferences

The patterns of correlation and partial correlation between the variables that were implied by our initial causal hypothesis (Fig 1) were significantly different than those in the empirical data, forcing us to reject this initial multivariate causal hypothesis. Our modified model (Fig 3) showed no statistical evidence of lack of fit but, since it was developed based partly on the empirical data themselves and since it has relatively low statistical power (only 25 species), it should be viewed as simply a plausible hypothesis that is consistent with the available evidence but that requires independent testing. However, the available evidence goes well beyond our specific data since there exist multiple studies reporting correlations between variables included in our model and that guided our modifications. Such correlative relationships do not justify by themselves the existence of the causal relationships shown in our model but are consistent the biological hypotheses leading to the relationships included in our path model. All the details on the correlations supporting the model are available in S2 Table.

The model shows strong direct and indirect relationships between soft and hard traits along with direct and indirect relationships between soft traits. Importantly, the model doesn’t show any direct relationship between a soft trait and habitat wetness. Instead, all of the links between soft traits and habitat affinities are indirect and mediated by the hard traits. The links between morphological traits are largely supported by key studies [29,30].

Three of the five soft traits (SLA, LDMC and LNC) are commonly used in functional ecology and are included in Wright et al.'s (2004) [30] leaf economic spectrum. The LDMC shows a negative causal link with LNC (R^2^ = 0.40). We found a negative causal link between SLA and LDMC as expected from the literature [29,30]. The stomatal area and specific root length are less connected to the other soft traits. Stomatal area is linked to only one other trait (LDMC), being negatively covariant, which is not consistent with Römermann et al. (2016) [37] who reported a small but positive correlation (R^2^ = 0.03). We know of no mechanistic reason why species whose leaves have greater tissue density (higher LDMC) would have smaller stomates, which is why we modelled this as a free covariance rather than a directed relationship.

SRL in not linked to any other soft traits. Note that these greater SRL values are not simply plastic responses that are caused by a drying soil because they are measured in soil maintained a field capacity. It is likely that species adapted to drier soils have a greater plasticity in SRL allowing them a greater proportional allocation to roots length than root biomass during drought in order to gain access to water in deeper soil layers during the dry season and therefore maintain photosynthetic rates [38]. This would be a case of drought avoidance rather than drought tolerance.

### Antagonistic and synergistic direct and indirect effects of soft traits on habitat preferences

Although our soft traits do not have any direct causal links to habitat affinities, they are all linked to habitat affinities. The overall correlations are weak precisely because (i) they act only indirectly through the mediating effects of the physiological traits and (ii) they often have qualitatively different effects via different physiological variables. In other words, our morphological traits have both antagonistic and synergistic effects at the same time on the physiological traits. The model does not show any link between soft traits and the habitat affinities that is not mediated by one or more hard traits. When we added direct links between soft traits and the habitat affinities in the model, the links were always non-significant and the model was rejected.

The path analysis allows us to investigate the reason why soft traits are only very weakly correlated to the habitat affinities. Mathematically, the correlation between two variables is equal to the sum of the product of each path coefficient between these two variables along each directed path linking them [21]. This means, by definition, that the correlation between soft traits and habitat will always be weaker than the correlation between hard traits and habitat whenever the link between soft traits and the habitat affinity is only indirect. Furthermore, whenever there is more than one directed path from a soft trait to the habitat, and when the signs (positive or negative) along these alternate paths are different, the overall correlation between a soft trait and the habitat will be further weakened despite the fact that these indirect causal paths exist and may even be individually strong (Fig 2).

For instance, if we consider the overall relationship between habitat wetness and leaf dry matter content, several paths exist from one to the other in Fig 2. One path goes from LDMC to habitat wetness via the maximum stomatal conductance. Regarding only the signs of the path, the link between LDMC and g_s_^max^ is positive and the link between g_s_^max^ and habitat wetness in positive. Multiplying the two gives an overall positive effect. Another path goes from LDMC to habitat wetness via leaf nitrogen content and g_s_^max^. The link between LDMC and LNC is negative, the link between LNC and g_s_^max^ is positive and the link between g_s_^max^ and habitat wetness is positive. Multiplying them together gives a negative effect along this path. One additional path goes from LDMC to habitat wetness via SLA, photosynthesis at wilting and water use efficiency at wilting. Multiplying the path coefficients along this path (Fig 3) gives a positive effect. Regarding only these three indirect effects linking LDMC and habitat affinities (there are others in Fig 3), we can see that there are both antagonistic and synergistic effects linking LDMC and an affinity for habitat wetness and this attenuates the overall correlation between these two variables.

The model we have proposed is based on experiments imposing an artificial drought and is performed on plants grown in individual pots. In such experimental conditions, individuals do not compete for resources (water, light, nutrients) and so such results are applicable to potential niches rather than to realize niches [1]. To obtain a more realistic model that could be applied to a population level, the model has to be tested in an experiment including inter- and intra-specific competition for water supply. In the context of gradients in soil water availability, these realized hydrological niches would be determined by trade-offs in functional traits that are involved in the water economy of a plant and that are implicated in determining plant performance along this environmental gradient.

The path analysis method used in this study allows us to better understand the complex relationships existing between soft traits, hard traits and habitat preferences. We think that the complexity of the relationships along with the antagonistic and synergistic effects between traits are the reason why the morphological soft traits are poor predictors of the habitat affinities despite the fact that the soft traits do affect affinities for soil moisture and so are functional. However, the functional significance and meaning of such soft traits are complicated by the intricate and indirect nexus of links between them and ecological distributions along environmental gradients. As stated before by Cornelissen et al. (2003) and Pérez-Harguindeguy et al. (2013) [2,14] and, we need to better assess the significance of the soft traits in relation to hard traits and the significance of hard traits in relation to plant performance or fitness.

## Supporting information

S1 Table Traits used in the analysis. Traits values are the mean of five individuals per species. Units are reported in Table 2.
(XLSX)

S2 Table References reporting correlations between variables included in our model (Fig 3). The columns "Link to" and "from" report the causal links presented in the path diagram (Fig 3). The "Path coefficient sign" column reports the sign of the causal link presented in the model. The last two columns report the information on correlations found in the cited literature, as well as the type of plant, species or model used. All references cited in the table are listed in S1 Text.
(XLSX)

S1 Text List of references cited in S2 Table. (DOCX)
