# Supplementary material for: Linking hard and soft traits: Physiology, morphology and anatomy interact to determine habitat affinities to soil water availability in herbaceous dicots
Source: PLoS One. 2018 Mar 28;13(3):e0193130. doi: 10.1371/journal.pone.0193130 (PMC5873933; doi:10.1371/journal.pone.0193130)
Supplement: S1 Text — (DOCX) [file pone.0193130.s003.docx]

**S1 Text. List of references cited in S2 Table.**

Alameda, D., and Villar, R. (2012). Linking root traits to plant physiology and growth in Fraxinus angustifolia Vahl. seedlings under soil compaction conditions. Environ. Exp. Bot. 79, 49–57.

Chaturvedi, R.K., Raghubanshi, A.S., and Singh, J.S. (2014). Relative effects of different leaf attributes on sapling growth in tropical dry forest. J. Plant Ecol. 7, 544–558.

Daly, E., Porporato, A., and Rodriguez-Iturbe, I. (2004). Coupled Dynamics of Photosynthesis, Transpiration, and Soil Water Balance. Part I: Upscaling from Hourly to Daily Level. J. Hydrometeorol. 5, 546–558.

Galmés, J., Ochogavia, J.M., Gago, J., Roldàn, E.J., Cifre, J., Conesa, M.À., Ochogavía, J.M., Gago, J., Roldán, E.J., Cifre, J., et al. (2013). Leaf responses to drought stress in Mediterranean accessions of Solanum lycopersicum: anatomical adaptations in relation to gas exchange parameters. Plant, Cell Environ. 36, 920–935.

Garnier, E., Navas, M.-L., and Grigulis, K. (2016). Plant functional diversity: organism traits, community structure, and ecosystem properties. (Oxford University press, Oxford, UK.).

Juhrbandt, J., Leuschner, C., and Hölscher, D. (2004). The relationship between maximal stomatal conductance and leaf traits in eight Southeast Asian early successional tree species. For. Ecol. Manage. 202, 245–256.

Kröber, W., and Bruelheide, H. (2014). Transpiration and stomatal control: A cross-species study of leaf traits in 39 evergreen and deciduous broadleaved subtropical tree species. Trees - Struct. Funct. 28, 901–914.

Mitchell, P.J., Veneklaas, E.J., Lambers, H., and Burgess, S.S.O. (2008). Leaf water relations during summer water deficit: Differential responses in turgor maintenance and variation in leaf structure among different plant communities in south-western Australia. Plant, Cell Environ. 31, 1791–1802.

Reich, P.B., Walter, M.B., Tjoelker, M.B., Vanderklein, D., and Buschena, C. (1998). Photosynthesis and respiration rates depend on leaf and root morphology and nitrogen concentration in nine boreal tree species differing in relative growth rate. Funct. Ecol. 12, 395–405.

Römermann, C., Bucher, S.F., Hahn, M., and Bernhardt-Römermann, M. (2016). Plant functional traits - fixed facts or variable depending on the season? Folia Geobot. 51, 143–159.

Schulze, E.D., Kelliher, F.M., Korner, C., Lloyd, J., and Leuning, R. (1994). Relationships among maximal stomatal conductance, carbon assimilation rate, and plant nitrogen nutrition: A global ecology scaling exercise. Annu. Rev. Ecol. Syst. 25, 629–660.

Tyree, M.T. (2007). Chapter 6 : Water Relations and Hydraulic Architecture. In Functional Plant Ecology, Second Edition, F.I. Pugnaire, and F. Valladares, eds. (CRC Press, New York, USA.), pp. 175–211.

Wright, I.J., Reich, P.B., Westoby, M., Ackerly, D.D., Baruch, Z., Bongers, F., Cavender-bares, J., Chapin, T., Cornelissen, J.H.C., Diemer, M., et al. (2004). The worldwide leaf economics spectrum. Nature 428, 821–827.
